# Understanding animal fears: a comparison of the cognitive vulnerability and harm-looming models

**DOI:** 10.1186/1471-244X-7-68

**Published:** 2007-12-01

**Authors:** Jason M Armfield

**Affiliations:** 1 Australian Research Centre for Population Oral Health, School of Dentistry, University of Adelaide, South Australia, Australia

## Abstract

**Background:**

The Cognitive Vulnerability Model holds that both clinical and sub-clinical manifestations of animal fears are a result of how an animal is perceived, and can be used to explain both individual differences in fear acquisition and the uneven distribution of fears in the population. This study looked at the association between fear of a number of animals and perceptions of the animals as uncontrollable, unpredictable, dangerous and disgusting. Also assessed were the perceived loomingness, prior familiarity, and negative evaluation of the animals as well as possible conditioning experiences.

**Methods:**

162 first-year University students rated their fear and perceptions of four high-fear and four low-fear animals.

**Results:**

Perceptions of the animals as dangerous, disgusting and uncontrollable were significantly associated with fear of both high- and low-fear animals while perceptions of unpredictability were significantly associated with fear of high-fear animals. Conditioning experiences were unrelated to fear of any animals. In multiple regression analyses, loomingness did not account for a significant amount of the variance in fear beyond that accounted for by the cognitive vulnerability variables. However, the vulnerability variables accounted for between 20% and 51% of the variance in all animals fears beyond that accounted for by perceptions of the animals as looming. Perceptions of dangerousness, uncontrollability and unpredictability were highly predictive of the uneven distribution of animal fears.

**Conclusion:**

This study provides support for the Cognitive Vulnerability Model of the etiology of specific fears and phobias and brings into question the utility of the harm-looming model in explaining animal fear.

## Background

Fear of animals is prevalent in many countries. In Australia, studies have found up to 40% of children suffer from fear of animals such as spiders and snakes [1] while in the United States of America 22% of older adults report fear of snakes and 13% report fear of dogs [2]. Specific Phobias, considered to be fears involving associated functional impairments [3], are less common but may still have a relatively high prevalence in some populations. In Israel, for example, 31% of a military population reported fear of animals, with approximately 20% of these people indicating phobic symptoms [4]. Reported fear of animals varies by age, sex, cultural background and geographic location of respondents. In many countries, dangerous animals such as snakes, spiders, sharks, wasps and crocodiles or alligators are relatively common. In Australia, for example, about 3000 people will be bitten by a snake in any one year [5] with bites from spiders being about four times more likely [6]. In India, poisonous snakes are estimated to kill 50,000 Indians a year [7]. Rare events such as deaths from shark or crocodile attacks and more common events such as people being mauled or killed by domesticated dogs [8] invariably attract considerable media attention in Western countries. All these factors serve to keep the danger of animals in peoples' minds.

Although fears and phobias of dangerous animals may be readily understandable, fear of non-dangerous animals is poorly accounted for in the literature. However, Armfield [9] has argued that fear is not merely a reaction to danger but that perceptions of control, predictability, and disgust are also crucial determinants of an individual's fear response. These variables are believed to be conceptually connected, with perceptions of uncontrollability, unpredictability, dangerousness, and disgustingness comprising a set of vulnerability cognitions which are central to the etiology of fear for a given stimulus.

The Cognitive Vulnerability Model neatly explains two traditionally vexing questions regarding the characteristics of fears and specific phobias. First, why is it that some people fear a given stimulus when others do not, despite apparently similar learning histories? According to the Cognitive Vulnerability Model, it is the perceptions of a stimulus as uncontrollable, unpredictable, dangerous and disgusting which directly determine fear of that stimulus. While learning experiences may help shape these vulnerability-related perceptions they are not causal per se.

The second question addressed by the Cognitive Vulnerability Model is what causes the uneven distribution of feared stimuli and situations in the population? Although the idea of biological preparedness [10], an inbuilt biological predisposition to fear some stimuli, was introduced to address this issue and is now widely regarded as a psychological truism [11], it does suffer from problems and is the source of some debate in the literature [12]. For example, the exact biological mechanisms underlying the notion of preparedness have never been identified, alternative hypotheses can readily explain preparedness-like effects obtained from laboratory experiments [13,14], and many of the key assumptions of the preparedness theory have not been supported [13]. De Jong and Merckelbach [12] have concluded that there is no convincing evidence to support the preparedness theory, while McNally has pointed out the difficulties inherent in testing possible evolutionary scenarios for preparedness effects [14]. In contrast to preparedness theory, the Cognitive Vulnerability Model predicts that the uneven fear distribution within a population is a direct result of differences in the perceived uncontrollability, unpredictability, dangerousness and disgustingness of the specific stimuli or situations. For instance, the higher prevalence of fears and phobias related to animals such as spiders, snakes, rats and sharks in comparison to moths, rabbits or cats is precisely a result of these animals being perceived as being more uncontrollable, unpredictable, dangerous and disgusting.

Some support for the Cognitive Vulnerability Model of the etiology of fear comes from a study by Armfield and Mattiske [15] which found strong correlations between the four vulnerability perceptions and fear of spiders. They also found that the vulnerability-related perceptions accounted for a significant amount of the variance in spider fears beyond that accounted for by a number of classical conditioning, vicarious, and informational learning events. Additionally, experimental studies manipulating perceptions of the uncontrollability, unpredictability and dangerousness of spiders were found to effect self-rated spider fear [16,17]. Support for the relationship between fear and the vulnerability variables also comes from a series of studies by Riskind and colleagues [18-20]. Riskind *et al.*[18] combined two items each for danger, probability of harm, imminence, uncontrollability, and unpredictability into a global index of threat cognitions. Although the independent effects of these variables were not explored, the Threat Cognitions Index (TCI), formed from the combination of these variables, was found to be highly correlated with fear of spiders. Similarly, Riskind *et al.*[20] found scores on the TCI to be significantly higher for high spider-fearful individuals than for low spider-fearful subjects.

Despite the consistency of the findings of Riskind *et al.*[18,20] with those of Armfield and Mattiske [15], the studies by Riskind and his colleagues were designed to investigate the "harm-looming" model of fears and specific phobias. Riskind has posited that the perception of "loomingness" is essential to the understanding of fear with fear stemming from a person's anticipation that a danger is rapidly moving closer [18]. This anticipation is assumed to involve perceptions of both velocity and acceleration [18,20]. It is claimed that individuals fear stimuli in direct proportion to their perception of such forward motion in these stimuli and not simply to the extent that the objects or events are perceived as heralding aversive consequences.

There is now an extensive body of research investigating the harm-looming model and the more recent extension dealing with looming maladaptive style [21,22]. Overall, the results of most of these studies have been consistent with the underlying premises of the harm-looming model. However, a closer examination of some of this work reveals some methodological and interpretive concerns. For example, in an initial series of studies by Riskind *et al.*[18], the findings which are taken to support the harm-looming model are also consistent with the idea that the relationship between loomingness perceptions and fear is spurious, resulting from high correlations between threat cognitions (comprising the vulnerability variables) and both spider fear and perceptions of loomingness. In addition, the series of studies is characterised by various methodological and reporting problems, statistical analyses are selectively reported and some potentially important analyses are not conducted. Finally, the items that comprise a number of the measures (e.g., uncontrollability, unpredictability, and danger) are not described, and the significant effect for loomingness found in Study 3 of the paper may well stem from the demand characteristics resulting from the use of a repeated measures design.

Demand characteristics may also affect the study by Riskind and Maddux [23], which attempted to experimentally manipulate both perceptions of loomingness and self-efficacy in relation to spiders. A more serious concern with this study, however, is the dubious relevance of its findings to the harm-looming model. In an attempt to manipulate perceived loomingness, subjects were shown film clips of a spider moving towards them, remaining stationary, and moving away from them. The manipulation check on the Motion conditions consisted of two questions which asked about the speed and the physical mobility of the spider. However, while these questions do relate to perceptions of motion, discussion of the statistical analyses were frequently couched in terms of perceptions of loomingness, despite the fact that perceptions of loomingness were not actually measured. Therefore, the conclusion by Riskind and Maddux that "experimentally induced perceptions of looming motion...influenced perceptions of fear" (p. 82) may be inaccurate. A similar problem affects the series of studies by Riskind and Wahl [24] where the reasons underlying the results can not be attributed to differences in the perception of loomingness because this concept was again not measured.

Another study taken to support the harm-looming model has been reported by Riskind and Maddux [19]. However, while perceived loomingness was moderately correlated with fear of HIV situations, the study also found perceived lack of control, perceived danger, perceived likelihood of harm, and unpredictability of the HIV to correlate moderately with fear of HIV situations. When collapsed into a global index of threat cognitions these variables, which effectively comprise the four central perceptual characteristics of the Cognitive Vulnerability Model, predicted 41% of the variance in HIV fear scores. Loomingness, however, only predicted 4.5% of the variance in fear of HIV after controlling for threat cognitions. These results are similar to those reported by Riskind *et al.*[18] where the perceived loomingness of spiders accounted for only 2.8% and 4.3%, in studies 1 and 2 respectively, of the variance in spider fear scores beyond the contribution of the threat cognitions measured in these studies. It is the case, therefore, that the relationships between perceptions of loomingness, the vulnerability-related perceptions, and fear require further investigation in order to tease apart the direction and importance of these effects.

While the harm-looming model continues to attract considerable attention in the literature on the etiology of fear, several other theories and models are also prominent. Neo-conditioning theories of fear acquisition assume an important place for the concept of latent inhibition [25,26] and have been used to account for the failure of many people to develop a fear following a traumatic experience with a stimulus situation. In general, people come to a new stimulus with a history of associations involving that stimulus [27] and it is believed that these associations are influential in determining the occurrence or non-occurrence of subsequent conditioning. Laboratory studies have demonstrated that when a stimulus is presented alone on a number of occasions, the ease with which fear (or indeed any other response) can subsequently be conditioned is impaired [28-30]. This effect of prior exposure is also known as latent inhibition [31].

Latent inhibition is well documented in the animal conditioning literature, having been demonstrated with a variety of animals using a number of indices of conditioning [29]. However, the results of human conditioning studies have generally proven equivocal. While some studies have demonstrated an effect for pre-exposure on subsequent fear [32-34] other studies have failed to find this effect [35,36]. Siddle and Remington [37] reconcile these findings by arguing that much of the research that has failed to demonstrate latent inhibition effects has not used appropriate control procedures necessary to establish stimulus specific and associative effects for pre-exposure. However, even when such procedures have been followed the effects produced have been weak relative to those found in the animal literature [37].

Non-experimental studies have also failed to find a strong relationship between prior familiarity and fear. Doogan and Thomas [38], for example, found that significantly more high dog fear than low dog fear subjects reported having had little or no previous experience with dogs. However, there were no differences in past experience between high and low fear children. The reason for the differences between adults and children are difficult to explain. Indeed, and while the study results may be explainable by the non-associative account offered by Menzies and colleagues [39-41], there appears to be little direct support for the proposal that latent inhibition can explain the absence of fears in people who have experienced a traumatic event with a stimulus but not acquired a fear of that stimulus. Nonetheless, some theories give a prominent role to the hypothesised effects of stimulus pre-exposure on fear acquisition so further investigation of this phenomenon is warranted.

One variable which is rarely studied in relation to fear is negative evaluation. It is possible that some animals are perceived negatively whereas other animals are seen more positively. The possible dimensions of evaluation are many. Animals might be seen as more or less ugly, intelligent, useless, soft, aggressive, desirable, spiteful, etc. Negative evaluation is a potentially important variable in fear research due to the possibility that many of the relationships found between fear and other variables are in fact spurious. If there is a relationship between fear and negative perceptions (generally) of the feared stimulus, the finding of a relationship between, for example, fear and unpredictability may occur merely because unpredictability is perceived as a negative characteristic. For this reason there is a need to examine the relationship between fear and other variables after controlling for the potentially confounding effect of negative evaluation.

Armfield and Mattiske [15] found strong associations between perceptions of uncontrollability, unpredictability, dangerousness and disgustingness and fear of spiders. However, while it is often assumed that findings relating to spiders are generalisable to other animals and may be extended to other objects and situations entirely, there is no reason why any findings regarding fear of spiders are immediately generalisable even to other animals, let alone to other types of fears. Even if the four cognitive vulnerability variables are, in fact, related to the fear of various animals, it may be that the associations between the vulnerability perceptions and fear are a function of the specific animal under investigation. That is, the perceptions of particular stimuli may vary along the dimensions of uncontrollability, unpredictability, dangerousness, and disgustingness such that any particular stimulus demonstrates a specific vulnerability profile. Although there are a number of reasons for studying spiders, there remains a need to investigate the relationship between the vulnerability variables and fear of other animals. This is especially so for rarely feared animals, which are for the most part ignored in animal fear research.

The current study tested several hypotheses. First, it was predicted that each of the vulnerability variables (uncontrollability, unpredictability, danger, and disgust) would be significantly positively correlated with subjective fear of a number of different types of animals. Second, it was hypothesised that the four vulnerability variables would account for a significant amount of the variance in fear of each of the animals beyond that accounted for by perceived loomingness and after controlling for gender, negative evaluation, and prior familiarity. Finally, it was proposed that perceptions of unpredictability, uncontrollability, dangerousness, and disgustingness across a number of animals would exhibit significant positive correlations with fear of those animals. That is, the Cognitive Vulnerability Model would be able to explain the uneven distribution of fear across a number of animals.

## Method

A total of 162 first-year university students in Adelaide volunteered for participation in the study. Sixty-five subjects were male and 96 were female, with one subject not identifying his/her gender. The ages of all participants ranged from 17 to 47 with a mean of 22.9 years (*SD *= 7.6).

The participants' thoughts concerning a number of aspects of eight different animals were assessed using a questionnaire largely derived from Armfield and Mattiske [15]. The eight animals selected for study represented four animals traditionally found to elicit high fear responses and four animals that generally elicit little fear. The specific animals were selected on the basis of studies by Davey [42] and Bennett-Levy and Marteau [43]. Using a United Kingdom population, Davey examined self-reported fears to common indigenous animals. The five most feared animals were snakes, wasps, rats, cockroaches, and spiders. The six least feared animals were rabbits, guinea pigs, squirrels, fish, cats, and ducks. In an attempt to cross-validate the choice of animals for the present study, these findings were compared to those of Bennett-Levy and Marteau who looked at fear and avoidance of 29 harmless animals and found the six most feared animals to be rats, cockroaches, jellyfish, spiders, slugs, and grass snakes. The only overlap with the least feared animals found by Davey was for rabbits and cats. As a result of these findings, snakes, rats, cockroaches, and spiders were selected as high-fear animals while rabbits, guinea pigs, cats and ducks were chosen to represent low-fear animals. All these animals are found in Australia.

Two versions of the questionnaire were used, each looking at four animals (two high-fear and two low-fear). One version investigated spiders, ducks, rabbits, and cockroaches while the other version used cats, snakes, rats, and guinea pigs. The two versions were used in order to reduce the amount of time required for completing the questionnaire. Participants were randomly allocated one of the two questionnaire versions.

The variables measured were subjective fear, perceptions of dangerousness, disgustingness, uncontrollability, and unpredictability, loomingness, negative evaluation, familiarity, and learning history. All the variables were measured using self-endorsing items with possible response scores ranging from 1 to 7. Each item required a rating for all four animals used in each questionnaire. The internal reliability coefficients for each scale for each animal are given in Table 1. Most scales demonstrated reasonable reliability, however negative evaluation showed relatively low internal consistency and the Loomingness scale had poor internal consistency in relation to the low-fear animals.

**Table 1:** Reliability coefficients for all scales for all animals

	Danger^a^	Disgust^a^	Looming.^b^	Negative Evaluation^a^	Uncont.^c^	Unpred.^c^
High-fear animals						
Spiders	0.86	0.78	0.67	0.67	0.81	0.73
Cockroaches	0.79	0.78	0.62	0.53	0.78	0.81
Snakes	0.80	0.84	0.65	0.69	0.93	0.77
Rats	0.81	0.88	0.53	0.66	0.88	0.75
Low-fear animals						
Ducks	0.68	0.68	0.53	0.53	0.66	0.75
Rabbits	0.70	0.72	0.48	0.56	0.66	0.72
Cats	0.71	0.76	0.35	0.69	0.77	0.75
Guinea Pigs	0.72	0.80	0.56	0.53	0.67	0.72

*Note: *All reliability coefficients represent standardised item alpha

^a ^4 items in scale; ^b ^5 items in scale; ^c ^3 items in scale

Following Geer [44], the participants were asked to indicate how much anxiety or fear they felt towards the different animals. A score of 1 was assigned to the response *None*, and the values 2 to 7 assigned respectively to points on the scale described as *Very Little*, *A Little*, *Some*, *Much*, *Very Much*, and *Terror*. This scale has been extensively cited [45] and has been found to have good psychometric properties and to be related to other personality measures [44].

Perceptions of dangerousness were assessed using four questions. Two of these questions referred to the potential dangerousness of the animal while the other two questions measured the subject's perceived likelihood of actually being harmed. In an attempt to obtain a general measure of perceived dangerousness for each animal the questions either referred to situations where the specific breed or genus of the animal was unknown or were related to thoughts concerning the majority of these animals. The four items used to measure disgust in this study consisted of a short version of an eight-item scale employed by Armfield and Mattiske [15]. This measure has two sub-scales relating to disgust-eliciting features such as appearance and feel and disgust in relation to disease or dirtiness. Two items related to each of these sub-scales. Perceptions of uncontrollability were assessed using three questions relating to general feelings of control when interacting with the particular animal. These questions were selected from a larger measure designed by Armfield and Mattiske [15] on the basis of an analysis of optimum reliabilities for minimum items. All subjects also completed a three-item measure of unpredictability of movement for each animal. Higher scores on each of the four scales indicated increased perceptions of dangerousness, disgustingness, uncontrollability and unpredictability respectively. A list of the questions for each vulnerability-related perception is provided in the Appendix.

Loomingness was measured using the five-item scale developed and employed by Riskind *et al.*[18]. An example item is "How slow or fast would a ______ move towards you?" This scale has been found to have good internal reliability (α = .93) and has subsequently been used in annotated format in other studies. Interestingly, the internal reliability coefficients of the Loomingness scale obtained for all animals in this study were appreciably less than those previously reported. Indeed, even using only two items from the loomingness scale, Riskind *et al.*[20] found a high internal consistency (α = .93) for the loomingness measure. Reliability coefficients in this study ranged from .35 to only .67 (Table 1).

Participants were asked four questions designed to measure negative evaluations of each animal. These evaluation items were obtained from the Semantic Differential Scale developed by Osgood, Suci, and Tannenbaum [46]. The four items were selected according to their relevance to possible evaluations of animals and comprised judgements of the intelligence, usefulness, cruelness, and friendliness of the animals. An estimate of the negative evaluation of each animal was obtained by taking the mean of the scores for each item. High scores represented a negative evaluation, low scores a positive evaluation, and scores in the middle of the possible range represented a neutral evaluation.

Familiarity was assessed by asking the participants how much personal experience they had had with each animal, with responses ranging from *Considerable experience *to *No personal experience*.

Six types of learning events were assessed, which may be classifiable as classical, vicarious, and informational conditioning experiences. These items required ratings of the worst harm/pain/illness ever inflicted on oneself, seen or heard about being inflicted upon another, the degree of mother's and father's fear, and the most fear ever seen being demonstrated by another person. This method of eliciting historical and experiential factors in the development of animal fears has been commonly used [47,48]. Unlike the Phobic Origin Questionnaire developed by Öst and Hugdahl [49] which used a yes/no response classification, the current study required subjects to make ratings on a 7-point scale representing either degree of harm (*None inflicted *to *Extremely serious*) or degree of fear (*No fear *to *Terrified*). An option of *Unknown *was provided for ratings of mother's and father's fear.

### Procedure

Ethical approval was obtained for the study and informed consent was obtained from the participants who were told that participation was voluntary, that they would not be individually identifiable and that they were free to both discontinue their participation at any time and to decline to answer any particular question. Participants were tested in a group format in a university setting and were given brief information regarding the questionnaire, being told that it concerned beliefs that may or may not be related to animals. In an attempt to reduce demand characteristics, the participants were not informed that the questionnaire related to fears of animals. In addition, items relating to uncontrollability, unpredictability, dangerousness, disgustingness, loomingness, negative evaluation, and familiarity were presented in a mixed order prior to the questions relating to fear and learning history.

### Analysis

Descriptive statistics were calculated for the fear, negative evaluation, perceptions of dangerousness, disgustingness, uncontrollability, unpredictability, loomingness, and familiarity associated with each of the eight animals. Pearson *R *correlations were used to assess the association between fear of each animal and the Independent Variables (IVs), including possible conditioning experiences. Two series of hierarchical multivariate regression models were constructed for fear of each animal. For one series, the cognitive vulnerability variables were entered as the last step, after controlling for perceived loomingness and other possible confounders. In the other series, perceived loomingness was entered as the last step after controlling for the cognitive vulnerability variables and other possible confounders. Statistical significance was assessed via the change in *R*^2^. Finally, aggregate data were used to determine the linear associations between the fear of animals and the various IVs. Because of the large number of analyses, the criterion alpha for rejecting the null hypothesis was set at 0.01 to reduce the risk of Type 1 error.

## Results

The descriptive statistics for the high-fear animals on all the measures are presented in Table 2. In general, mean fear for these animals ranged from a rating of 3.00 for cockroaches, to 4.47 for snakes. Snakes were rated as being the most dangerous, uncontrollable, unpredictable, and looming of the animals. Cockroaches were perceived as the most disgusting animal and were evaluated more negatively than the other high-fear animals, although they were rated as the least dangerous, uncontrollable, and unpredictable. Spiders were the most familiar of the high-fear animals (mean = 4.76) while snakes and rats were the least familiar (means = 2.73 and 2.81 respectively).

**Table 2:** Descriptive statistics for all measures for high-fear animals

	Spider^a^		Cockroach^a^		Snake^b^		Rat^b^	
				
	Mean	SD	Mean	SD	Mean	SD	Mean	SD
Fear	4.00	1.77	3.00	1.86	4.47	1.83	3.44	1.94
Dangerousness	3.93	1.49	2.16	1.11	5.04	1.22	3.20	1.31
Disgustingness	4.94	1.47	6.01	1.18	4.14	1.66	5.10	1.70
Uncontrollability	3.44	1.85	2.71	1.68	4.89	1.82	3.57	1.86
Unpredictability	4.57	1.43	4.16	1.66	4.95	1.42	4.49	1.33
Loomingness	4.34	1.22	3.82	1.11	4.83	1.18	4.23	1.00
Negative evaluation	3.98	1.11	4.81	1.05	4.23	1.13	4.17	1.17
Familiarity	4.76	1.69	3.99	1.87	2.73	1.87	2.81	1.79

^a ^For all measures, *n *= 88

^b ^For all measures, *n *= 90

As expected, all the low-fear animals were feared less than the high-fear animals (Table 3). All fear ratings were between *None *and *Very Little *on average. Of the low-fear animals, ducks were the most feared (mean = 1.67) while rabbits were the least feared. Guinea pigs were perceived as the most disgusting and unpredictable of the low-fear animals and were evaluated the most negatively. Ducks were rated as the most uncontrollable of the low-fear animals and cats obtained the highest rating of loomingness and dangerousness.

**Table 3:** Descriptive statistics for all measures for low-fear animals

	Duck^a^		Rabbit^a^		Cat^b^		Guinea Pig^b^	
				
	Mean	SD	Mean	SD	Mean	SD	Mean	SD
Fear	1.67	0.91	1.40	0.74	1.51	0.84	1.52	0.93
Dangerousness	1.78	0.75	1.60	0.72	1.98	0.80	1.83	0.87
Disgustingness	3.09	1.08	3.02	1.11	2.16	1.71	3.61	1.37
Uncontrollability	1.84	0.92	1.55	0.81	1.71	0.98	1.73	0.98
Unpredictability	3.37	1.29	3.26	1.38	3.22	1.40	3.41	1.34
Loomingness	3.39	0.85	3.43	0.87	3.78	0.78	3.19	0.92
Negative evaluation	3.24	0.91	3.50	1.05	2.96	1.23	3.80	0.97
Familiarity	4.40	1.94	4.69	1.91	6.11	1.55	3.16	2.11

^a ^For all measures, *n *= 88

^b ^For all measures, *n *= 90

All the low-fear animals were rated as less dangerous, disgusting, uncontrollable, and unpredictable than the high-fear animals and were evaluated less negatively. Although the low-fear animals were rated as less looming than the high-fear animals there was little difference between cats (a low-fear animal) and cockroaches (a high-fear animal). Finally, although cats were rated as the most familiar of any of the eight animals, spiders (a high-fear animal) were rated as more familiar than the remaining low-fear animals, and cockroaches were rated as more familiar to participants than were guinea pigs.

All of the vulnerability variables were significantly correlated with fear of each of the high-fear animals (Table 4). Uncontrollability consistently exhibited the highest correlations with fear while unpredictability had the lowest correlations with fear. Perceived dangerousness, disgustingness, and uncontrollability were also significantly correlated to fear of each of the low-fear animals. Once again, perceptions of uncontrollability had the highest correlations with fear of each of the animals. However, the only significant correlation between unpredictability and fear for the low-fear animals was for cats. For ducks, rabbits, and guinea pigs perceptions of unpredictability were not significantly related to fear.

**Table 4:** The relationship between fear of each animal and dangerousness, disgustingness, uncontrollability and unpredictability

	Dangerousness	Disgustingness	Uncontrollability	Unpredictability
High-fear				
Spiders	0.70**	0.58**	0.85**	0.40**
Cockroaches	0.46**	0.49**	0.82**	0.32*
Snakes	0.71**	0.60**	0.83**	0.57**
Rats	0.77**	0.66**	0.88**	0.58**
Low-fear				
Ducks	0.60**	0.30*	0.72**	0.24
Rabbits	0.62**	0.34**	0.74**	0.25
Cats	0.47**	0.32*	0.73**	0.35**
Guinea Pigs	0.38**	0.40**	0.51**	0.13

* *p *< 0.01; ** *p *< 0.001

Perceptions of loomingness were significantly, albeit moderately, correlated with fear of each of the high-fear animals (Table 5). However, the only significant correlation between loomingness and fear for the low-fear animals was for rabbits. It was expected that people who are afraid of an animal may evaluate the animal negatively, perhaps as a consequence of their fear. Consistent with this prediction, negative evaluation was significantly correlated with fear of the high-fear animals. However, the only animal showing a significant correlation between fear and negative evaluation for the low-fear animals was ducks. In general, there were few significant relationships between prior familiarity with an animal and fear of that animal. However, for both snakes and rats familiarity exhibited a statistically significant negative correlation with the fear measure. That is, the more familiarity with the animal, the less fear indicated.

**Table 5:** The relationship between fear of each animal and loomingness, negative evaluation and familiarity

	Loomingness	Negative Evaluation	Familiarity
High-fear			
Spiders	0.41**	0.40**	-0.20
Cockroaches	0.31*	0.42**	0.17
Snakes	0.41**	0.58**	-0.42**
Rats	0.41**	0.57**	-0.41**
Low-fear			
Ducks	0.08	0.43**	-0.02
Rabbits	0.28*	0.16	-0.09
Cats	0.11	0.17	-0.10
Guinea Pigs	0.23	0.19	-0.19

* *p *< 0.01; ** *p *< 0.001

Table 6 shows the Pearson *R *correlations between fear and potential conditioning experiences for each animal. Overall, there were very few statistically significant associations. Only one association between fear of high-fear animals and possible learning experiences reached statistical significance, while no significant associations were evident for low-fear animals.

**Table 6:** Correlations between fear of each animal and ratings of possible conditioning experiences

	Exp. 1	Exp. 2	Exp. 3	Exp. 4	Exp. 5	Exp. 6
High-fear animals						
Spiders	-0.09	0.06	0.09	0.01	-0.11	0.01
Cockroaches	0.24	0.11	0.06	0.01	-0.08	0.04
Snakes	0.05	0.04	0.05	0.04	0.14	0.02
Rats	-0.04	0.09	0.12	0.11	0.22	0.25*
Low-fear animals						
Ducks	0.10	0.08	0.13	0.17	0.15	0.06
Rabbits	0.07	0.05	0.18	0.21	0.11	0.17
Cats	0.18	0.12	0.15	0.24	0.21	0.14
Guinea Pigs	0.24	0.20	0.25	0.13	0.15	0.16

* *p *< 0.01

In order to test the effects of the vulnerability variables on fear after controlling for the other variables, a series of hierarchical multiple linear regression analyses were conducted. For each animal, the vulnerability variables were entered as a block at Step 3, following the forced entry of gender and negative evaluation at Step 1 and loomingness at Step 2. Due to its non-significant effect on fear of most of the animals used in this study, Familiarity was not used in the regression equations. In addition to testing for the independent effects of the vulnerability variables on each animal, the independent effect of loomingness was also determined. Once again, a series of hierarchical multiple linear regression analyses were conducted, this time with gender and negative evaluation at Step 1, uncontrollability, unpredictability, dangerousness, and disgustingness at Step 2, and loomingness at Step 3. These analyses were done for each animal using fear as the DV. Because of the correlations between many of the variables, checks for multicollinearity were carried out as recommended by Tabachnick and Fidell [50]. However, no evidence of multicollinearity was found among the variables.

A summary of the full series of multiple regressions is given in Table 7. For each animal, the vulnerability variables accounted for a significant amount of variance in fear beyond that accounted for by gender, negative evaluation and loomingness. Loomingness, however, did not account for a significant amount of variance in fear, for any animal, beyond the variance accounted for by gender, negative evaluation, and the vulnerability-related variables. In general, there was either no effect or only a small independent effect for loomingness beyond that for the other variables.

**Table 7:** *R*^2 ^Change for independent contributions of loomingness and the vulnerability variables to fear of each animal

	Vulnerability variables^a^	Loomingness^b^
High-fear animals		
Spiders	0.50***	0.00
Cockroaches	0.47***	0.00
Snakes	0.35***	0.00
Rats	0.35***	0.01
Low-fear animals		
Ducks	0.41***	0.00
Rabbits	0.51***	0.00
Cats	0.50***	0.01
Guinea Pigs	0.20***	0.01

* *p *< 0.01. *** *p *< 0.0001

^a ^*R*^2 ^Change after controlling for Gender, Negative Evaluation, and Loomingness

^b ^*R*^2 ^Change after controlling for Gender, Negative Evaluation, Uncontrollability, Unpredictability, Dangerousness, and Disgustingness

Aggregated data were used to examine the linear associations between fear of the animals used in this study and perceptions of the animals as dangerous, disgusting, unpredictable, uncontrollable, looming, negatively evaluated and familiar. The associations with fear were strongest for unpredictability (*R*^2 ^= 0.98) and uncontrollability (*R*^2 ^= 0.94) while dangerousness and loomingness were also good predicators of fear (Table 8). The relationships of disgustingness and negative evaluation with fear did not reach statistical significance at the 0.05 criterion alpha. The associations between familiarity and fear were not statistically significant.

**Table 8:** Fear of animals and ratings of dangerousness, disgustingness, uncontrollability, unpredictability, loomingness, negative evaluation and familiarity

	Fear		
	
	*R*^2^	*p*	Beta
Dangerousness	0.87	0.001	0.935
Disgustingness	0.48	0.058	0.691
Uncontrollability	0.94	0.000	0.967
Unpredictability	0.98	0.000	0.993
Loomingness	0.87	0.001	0.931
Negative evaluation	0.47	0.062	0.683
Familiarity	0.24	0.222	-0.486

## Discussion

It was hypothesised that perceptions of an animal as uncontrollable, dangerous, disgusting, and unpredictable would be significantly related to fear of the animal. This prediction follows directly from the assumptions of the Cognitive Vulnerability Model [9]. This study found moderate to very high correlations between the four cognitive vulnerability variables and fear of high-fear animals. In addition, all the variables except unpredictability exhibited significant associations with fear of low-fear animals.

One of the main objectives of this study was to determine whether the perceptions of an animal as uncontrollable, unpredictable, dangerous and disgusting would explain a significant amount of variance in fear beyond the variance accounted for by the perceived loomingness of the animals. Riskind and colleagues have received much attention in regards to their looming vulnerability formulation of anxiety and the concept of a 'looming maladaptive style' [51]. In this study it was found that while the perceived loomingness of animals accounted for no more than 1% of the variance in fear for most animals beyond the variance explained by the vulnerability variables, the vulnerability variables accounted for between 20% and 51% of fear beyond that accounted for by perceived loomingness. The results provide strong support for the Cognitive Vulnerability Model of the etiology of fear and bring into question the utility of the harm-looming model. It is highly plausible that the concept of loomingness is in fact merely an aspect of an object's or animal's perceived unpredictability. There may also be a sense of loss of control in regards to a rapidly approaching stimulus. While loomingness may tap into perceptions of unpredictability and uncontrollability, it appears that the four cognitive vulnerability variables make a much more substantial contribution to explaining fear of animals than does the concept of perceived loomingness.

One result from this study that should be noted, however, and which may bear on the poor relationship between loomingness and fear, is that the internal reliability coefficients of the five-item Loomingness scale for all animals investigated in this study were much less than those reported previously. The discrepancies between previous studies and the current study are difficult to reconcile, but might go some way towards accounting for the weaker relationships between loomingness and fear found here.

Although classical conditioning theory would predict that increased familiarity with a stimulus would lead to a reduced likelihood of fear of the stimulus, no relationship between familiarity and subjective fear was found in the current study. Interpreted in light of the Cognitive Vulnerability Model this is understandable in that any significant relationships between familiarity and fear would stem not from the processes of habituation or reduced predictiveness but from the differential perceptions of uncontrollability, unpredictability, dangerousness, and disgustingness associated with each stimulus. That is, familiarity with an animal would be accompanied by various vulnerability perceptions of the animal that, in turn, would be related to fear. However, the pattern of these relationships would be complex. Familiarity with animals which might be considered as objectively unpredictable, uncontrollable, dangerous, and disgusting would be more likely to result in increased fear while familiarity with animals which are less likely to be perceived in such ways should result in less fear. However, familiarity with any given stimulus would lead to varying perceptions of the animal depending upon the specific experiences. It is not necessarily the case, therefore, that prior familiarity would automatically lead to reduced fear.

One of the enduring problems in fear research is the inability to account for the uneven fear distribution in the population. Population-based fear surveys demonstrate that some objects or situations are feared more than others [52]. An early attempt to account for this phenomenon used conditioning theory. According to this theory, those objects or situations most feared were also most liable to cause injury or pain and occurred most often. The high prevalence of fear of dangerous animals or situations seemed to lend credence to this argument. However, some researchers pointed out that many more injuries or painful experiences resulted from electric shocks, getting hit on the thumb by a hammer or falling down stairs than were received through spider or snake bites [13], let alone through such highly feared situations as nuclear war or terrorist attack. In the current study, the lack of significant associations between fear of animals and the experience of conditioning or learning events is testimony to the problems with the conditioning theory of fear acquisition. The reality is that many people have never had an adverse experience with the object of their fear or phobia [41].

Preparedness theory was developed to explain the uneven fear distribution, proposing that there is a biological preparedness or an inherent predisposition to learn to fear some stimuli more than others [10]. The theory holds that animals or situations which in pre-technological times have been associated with pain or injuries are more likely to be feared in the population today as a result of the increased propensity for fear learning to occur to these stimuli. Hence, dangerous animals, water, heights, and other stimuli perilous to pre-technological people should be more feared than modern day dangers such as guns, electricity outlets, or hammers. There is some support for this theory given that perceptions of the dangerousness of animals in this study were significantly related to fear of the animal despite the finding that very few people had had an aversive experience with any feared animal. However, the strongest predictors of fear, and therefore the best predictors of the uneven fear distribution, at least in terms of animals, were perceptions of the unpredictability and uncontrollability of the animals, accounting for 98% and 94% respectively of the variance in the distribution of animal fears. This is a quite remarkable finding, indicating that perceptual characteristics of animals almost perfectly predict the uneven distribution in animal fears. Of course, while the animals used in this study had a reasonable spread across the fear continuum it would be interesting and necessary to undertake similar analyses using an even larger number of animals to determine if the relationships found in this study continue to hold.

One of the weaknesses of this study is its reliance on the often used method of self-report. For example, judgements of familiarity and fear ratings are based on subjective reporting and these have several inherent weaknesses, including the possibility for memory biases and cognitive distortions. Adding behavioural assessments in future studies would be beneficial, although as the number of animals or situations being assessed increases this could clearly pose problems with behavioural assessment methodologies.

Although this study ostensibly deals with normative fears and no attempt was made to diagnose or classify any fearful people as having a Specific Phobia, the applicability of the Cognitive Vulnerability Model is not limited to sub-clinical manifestations of fear. It is generally regarded that fear is a uni-dimensional emotion with the designation phobia merely occupying an extreme position along the continuum. For example, Specific Phobia is defined by the American Psychiatric Association as a marked and persistent fear [3]. Perceptions of a given feared stimulus as uncontrollable, unpredictable, dangerous and disgusting are just as important in the etiology and maintenance of acute phobic fear as they are in sub-clinical fears. An understanding of these fundamental relationships will have important implications not just in understanding the causes of fear but for the treatment of phobic individuals. Cognitive-behavioural therapies should ideally focus on a person's perception of the uncontrollability, unpredictability, dangerousness and disgustingness of the feared stimulus. By addressing these core components of the vulnerability schema underlying the fear, reductions in fear should occur more rapidly and be longer lasting.

## Conclusion

In summary, this study found strong support for the proposition that the variables of uncontrollability, unpredictability, dangerousness and disgustingness are highly related to self-rated animal fears and that these variables strongly predict the uneven fear distribution of animals. Familiarity with animals and previous conditioning experiences were not related to fear of the animals. Finally, perceptions of the loomingness of the animals, although related to fear, failed to explain variance in fear of the animals beyond that accounted for by the vulnerability variables of uncontrollability, unpredictability, dangerousness and disgustingness. This study underlines the utility of investigating cognitive processes in relation to the fear experience and provides strong support for the Cognitive Vulnerability Model of the etiology of fear.

## Competing interests

The author(s) declare that they have no competing interests.

## Authors' contributions

JMA conceived, researched, performed the statistical analysis, and wrote this manuscript.

## Appendix

### Disgust

1. I think that _____ would feel pleasant to touch.

2. If I touched a _____ it would be important for me to wash my hands afterwards.

3. I think that _____ are dirty or unclean animals.

4. I would be revolted or disgusted if a _____ came into contact with my skin.

### Uncontrollability

1. I believe that I would be able to deal effectively with a _____ by myself when encountered.

2. If a _____ was nearby I would not feel in control of the encounter.

3. I do not believe that I would lose control over my actions in any way (e.g. panic or freeze) if a _____ came as rapidly as it could towards me.

### Unpredictability

1. I find most _____ to be predictable in their movements.

2. I think that the movement of _____ can be guessed in advance.

3. I never know what a _____ is going to do.

### Dangerousness

1. How potentially dangerous do you think that most _____ are to you?

2. I believe that if I came into contact with an unknown _____ I would be harmed.

3. I think that the majority of _____ are harmless to me.

4. I think that if I encountered an unknown _____ I would not be harmed in any way.

## Pre-publication history

The pre-publication history for this paper can be accessed here:


